# Fine-grained image classification using the MogaNet network and a multi-level gating mechanism

**DOI:** 10.3389/fnbot.2025.1630281

**Published:** 2025-08-06

**Authors:** Dahai Li, Su Chen

**Affiliations:** ^1^School of Electronics and Electrical Engineering, Zhengzhou University of Science and Technology, Zhengzhou, China; ^2^Department of Mechanical and Electrical Engineering, Henan Vocational College of Water Conservancy and Environment, Zhengzhou, China

**Keywords:** fine-grained image classification, MogaNet network, multi-level gating mechanism, feature elimination strategy, loss function

## Abstract

Fine-grained image classification tasks face challenges such as difficulty in labeling, scarcity of samples, and small category differences. To address this problem, this study proposes a novel fine-grained image classification method based on the MogaNet network and a multi-level gating mechanism. A feature extraction network based on MogaNet is constructed, and multi-scale feature fusion is combined to fully mine image information. The contextual information extractor is designed to align and filter more discriminative local features using the semantic context of the network, thereby strengthening the network’s ability to capture detailed features. Meanwhile, a multi-level gating mechanism is introduced to obtain the saliency features of images. A feature elimination strategy is proposed to suppress the interference of fuzzy class features and background noise. A loss function is designed to constrain the elimination of fuzzy class features and classification prediction. Experimental results demonstrate that the new method can be applied to 5-shot tasks across four public datasets: Mini-ImageNet, CUB-200-2011, Stanford Dogs, and Stanford Cars. The accuracy rates reach 79.33, 87.58, 79.34, and 83.82%, respectively, which shows better performance than other state-of-the-art image classification methods.

## 1 Introduction

Fine-grained image classification is a crucial task in the field of computer vision (CV), aiming to solve the problem of subdividing subclasses within the same general class (He and Peng, 2017). Fine-grained image classification has garnered the attention of researchers due to its wide-ranging application potential in areas such as smart transportation (Ke and Zhang, 2020), smart retail, and biodiversity monitoring. However, the uniqueness of fine-grained image classification lies in the significant differences within a class and the small differences between classes, which require the model to extract more subtle feature representations to achieve accurate image classification (Zhang W. et al., 2024). Therefore, compared with traditional image recognition tasks, fine-grained image classification is more challenging (Ke et al., 2023).

According to the type of label used, existing fine-grained image classification methods can be divided into two categories: one is a strong supervision method that relies on additional manual annotation information, and the other is a weak supervision method that relies only on image-level labels. The majority of early research focused on strong supervision methods. For example, Liu et al. (2016) proposed a Fully Convolutional Network (FCN) based on image masks. During the training process, the model accurately localized the head and torso of the object using manually marked key point information and then generated an image mask to exclude convolutional descriptors that contained the object region. Finally, the pooling operation integrated the features of the entire object, including the head and trunk, to achieve efficient classification. However, strong supervision methods require a significant amount of annotation information, such as object bounding boxes and key point information. The acquisition of this annotation information usually consumes a considerable amount of time and energy. Therefore, the method based on weak supervision has gradually become a new focus of research. Progressive multi-granularity training (PMG), proposed by Du et al. (2020), is a representative of the weak supervision method. The method utilizes the Jigsaw Puzzle Generator Module (JPGM) to segment the original image into blocks of different granularities and rearranges them into the network. In view of the sensitivity of the shallow layer network to the texture details of the object, PMG inputs fine-grained image blocks into the shallow layer network to extract the features of the object detail region. For coarse-grained image blocks, they are fed into the deep network to capture high-level semantic information of the image. Thus, the feature information of different granularities can be effectively fused, thereby improving the classification effect.

The early research is mainly based on convolutional neural network (CNN) technology to design fine-grained image classification models, and remarkable results have been achieved. However, due to the inherent limitation of the convolution operation, namely the receptive field constraint, these models tend to pay too much attention to local image details, and their global modeling ability for objects is relatively limited, which gradually leads to a performance bottleneck. At the same time, with Transformer excelling in the field of natural language processing, its powerful global information modeling capability has garnered widespread concern. Researchers have begun to introduce the vision transformer (ViT) (Yin et al., 2022) into various computer vision tasks, including pedestrian re-identification, object detection, and image classification, and have made positive progress. However, unlike other visual tasks, fine-grained image classification relies mainly on subtle inter-class differences. It therefore requires the network to be able to discover more subtle discriminative regional features. On the other hand, the ViT model relies on multi-head self-attention (MSA) to mine the correlation between each image block, paying more attention to the integration of global information and less attention to local key areas (Yuan et al., 2021). Therefore, applying the ViT directly to fine-grained image classification tasks may result in poor classification performance.

In response to the above problems, the researchers aim to improve the ViT, seeking to maintain its strong global modeling capabilities while enhancing its ability to capture local features, thereby improving ViT’s performance in fine-grained image classification tasks. For example, to address the ViT’s limitation in ignoring local regional information, Zhao et al. (2024) proposed a TransFG network comprising a Part Selection Module (PSM) and Contrastive Feature Learning (CFL).

The model used the PSM module to integrate the self-attention weights from the first 11 layers of the ViT to obtain the final attention map and then selected the PatchToken containing the locally important region of the object from the last layer of the self-attention encoding, compensating for the lack of detailed features in the network. In addition, the CFL module instructed the network to learn the characteristics shared among the same species and the differences between different ones, thereby amplifying the inter-class differences and narrowing the intra-class differences. Although TransFG considered the importance of local discriminative features to the network, it ignored the local, low, and high-level information contained in different levels, resulting in a partial loss of discriminative information. Therefore, Wang et al. (2021) proposed a new FFVT network based on TransFG. It filtered the most critical tokens in each self-attention encoding layer based on the size of the self-attention weight and integrated them. This method effectively integrated the subtle clues from different regions of the object and enriched the discriminant information of the network at different levels. In addition, to help the network identify the most discriminative area in the image, Xu et al. (2023) clipped and scaled the object area in the original image according to the attention weight and re-input it into the network, thereby improving the network’s recognition ability for images of different scales. Moreover, they also adopt distillation learning, which utilizes the features extracted from the CNN network to guide the ViT network in learning multi-source semantic information from different structures, thereby improving the network’s generalization ability.

Although existing ViT-based methods have made significant progress, several problems remain to be addressed. First, since images need to be divided into image blocks of a certain size before being input into the ViT network, and the size of these image blocks remains constant throughout the entire forward propagation process, the network can only process image information of the same granularity. However, for fine-grained image classification, features of different granularities have varying identification capabilities within the network, so the information learned by the network under a single granularity is incomplete and insufficient. Second, in fine-grained image classification, existing methods based on distillation learning often rely on a teacher model with a large number of parameters to guide a student model with a small number of parameters. However, due to the differences between the models, the teacher network with a better model effect is not necessarily conducive to the learning of the student network (Lu and Han, 2024); that is, the “knowledge” in the teacher network cannot be transferred to the student network. When the performance difference between the teacher network and the student network is too large, the guidance provided by the teacher network can lead to contradictions in the student network’s optimization. Third, different layers of the network have distinct areas of concern for the object; that is, each encoding layer has a different degree of influence on the network classification results. However, the existing method (Wan et al., 2021) simply fuses the self-attention weights of all layers when mining local details, thereby ignoring the differences between each self-attention encoding layer, which affects image classification. In addition, these methods only improve the large prediction region generated by the network, paying more attention to global information and paying less attention to local and underlying features. Background noise in complex scenes can significantly impair the network’s ability to focus on subtle local features.

Based on the above analysis, this study proposes a novel fine-grained image classification method utilizing the MogaNet network and a multi-level gating mechanism. First, using MogaNet (efficient multi-order gated aggregation network) as the backbone network, in the training stage, to extract multi-scale features, a contextual information extractor (CIE) is designed to remove the redundant information of the context, extract fine-grained local features with spatial context from the deep features, and capture the subtle changes in each region. Second, the feature elimination strategy (FES) is designed to mitigate the impact of fuzzy class features and backgrounds on classification, thereby improving the quality of processing ambiguous objects and complex backgrounds. Meanwhile, a multi-level gating mechanism is introduced to obtain the saliency features of images. Finally, features of different depths are fused to produce a refined prediction region. Experiments demonstrate that the proposed method exhibits favorable classification characteristics.

## 2 Related research

### 2.1 Fine-grained image classification

Currently, fine-grained image classification methods encompass local localization, feature coding, and attention mechanisms. For example, Ge et al. (2019) proposed a weakly supervised supplementary component model for fine-grained image classification. This method extracted rough object instances through mask R-CNN and CRF segmentation, then searched for the best component model, and fused this component information using bidirectional LSTM, which significantly improved classification performance. In order to obtain highly identifiable local discriminant regions, Liu et al. (2021) proposed a multi-regional attention method. The attention mechanism was repeatedly applied to focus on secondary features, combined with background removal and up-sampling techniques to obtain accurate local image features. Finally, a rectangular box was used to count positions, thereby reducing detail loss. In addition, combining multiple methods is also an effective strategy for improving performance and solving problems more comprehensively. Zhang et al. (2016) proposed a multi-scale collaborative differential network. They employed different kernels and pooling methods to obtain images of varying scales and then input these images into the basic network to generate multi-scale feature blocks. These blocks were fused to create features with more comprehensive information. Finally, these features were fed into a collaborative differential network to capture second-order information about the image. However, the effectiveness of the model largely depends on the adequacy of the data, and it is challenging to obtain data for rare species. Feature learning plays a crucial role in nearly all image tasks, including retrieval, detection, and classification. The performance of deep convolutional networks is particularly outstanding, and their success is mainly attributed to the ability to learn discriminative deep features. In the early days of deep learning development, the features (activations) of fully connected layers were typically used as image representations. Subsequently, with the continuous development of deep learning, researchers discovered that the feature maps of deeper convolutional layers contain intermediate and advanced information, such as parts of an object or the entire object. This has led to the widespread use of convolutional features/descriptors. Moreover, compared with the output of the fully connected layer, the application of encoding techniques to these local convolutional descriptors has brought significant improvements.

To some extent, the improvement of encoding techniques comes from the high-order statistics encoded in the final features. Especially for fine-grained recognition, when the Fisher vector encoding of SIFT features (Scale-Invariant Feature Transform) outperforms the fine-tuned AlexNet in several fine-grained tasks, the demand for end-to-end modeling of high-order statistics becomes apparent. Bilinear convolutional neural networks represent images as the mixed outer product of features derived from two deep convolutional neural networks, thereby encoding the second-order statistics of convolutional activation and significantly improving fine-grained recognition. However, bilinear mixed features generate extremely high-dimensional features, resulting in a significant increase in the number of parameters in the deep network classification module. This may lead to overfitting and make it impractical in practical applications, especially in large-scale applications. Therefore, this study focuses on exploring fine-grained image classification methods in a few-shot learning environment.

### 2.2 Few-shot learning

In a few-shot learning environment, due to the limited number of samples, the model is prone to overfitting, which reduces its generalization ability to new samples (Song et al., 2023). Few-shot learning strategies can be divided into two main categories: optimization-based meta-learning methods and metric-based learning methods. The core of meta-learning lies in enhancing the ability of models to adapt quickly to new tasks, although challenges arise when dealing with domain shifts. Metric learning is another widely used strategy that measures similarity by calculating the distance between sample features. For example, the prototype network proposed by Xu et al. (2020) calculates the average vector of samples within a class as the class prototype and compares the new sample with the class prototype to achieve fast classification. Yan et al. (2023) proposed the discriminant spatial metric network, aiming to maximize inter-class dispersion and minimize intra-class dispersion, and effectively use the geometric structure of samples to enhance the discriminant ability. Wei et al. (2019) introduced the concept of few-shot fine-grained recognition and designed a piecewise smart mapping function to analyze bilinear features by generating decision boundaries through a set of sub-classifiers. Folkesson et al. (2008) proposed a method for the adaptive selection of representative local descriptors, modifying the traditional KNN classification model to adjust the weights based on the distance between neighboring points. In addition, other studies (Nie et al., 2023) have used attention mechanisms to enhance semantic information and focus on image-rich regions.

### 2.3 Frequency domain learning

As an effective tool, frequency analysis has been widely utilized in deep learning to extract frequency features from signals. Mitsuhata et al. (2001) employed the Fourier transform and its inverse to reduce the discrepancy between the source distribution and object distribution by adjusting the low-frequency components. Zhong et al. (2022) converted RGB color images into the frequency domain and developed a frequency selection strategy to select frequencies with abundant information. In addition, Chen and Wang (2021) discussed the influence of different frequency components on few-shot learning tasks and proposed a new frequency-guided few-shot learning framework. The framework could adaptively mask relevant information in images according to specific task requirements. Unlike the above work, this study identifies the overall structure of the image by extracting features in the frequency domain, thereby avoiding disturbances from local noise. Local changes in the image are then captured in the airspace. The combination of the two provides better resistance to noise and other interference, and parallel processing can also provide higher degrees of freedom for subsequent operations.

## 3 Proposed image classification

**Figure 1:**
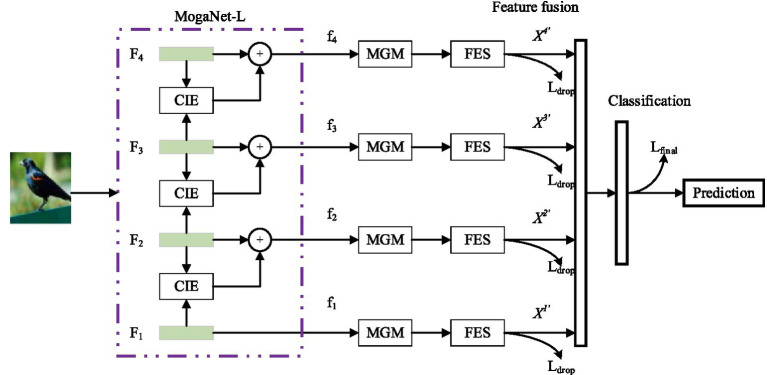
Proposed image classification structure.

The proposed fine-grained image classification method, based on the MogaNet network and a multi-level gating mechanism, is illustrated in Figure 1.

First, the MogaNet backbone network is utilized to extract multi-scale feature maps. Then, a contextual information extractor is proposed to extract both global features, including shapes and textures, and local features, including edges and corners, using multi-scale sliding windows. The attention mechanism is used to suppress low-context areas and enhance the ability to capture detailed features. Meanwhile, a multi-level gating mechanism (MGM) is introduced to obtain the saliency features of images. Then, a feature elimination module is designed to reduce the impact of fuzzy class features and background features on network classification by combining multi-modal feature interaction. Finally, a full connection layer is used to fuse features of different scales, and different input images are classified through the classification layer.

### 3.1 MogaNet

The backbone network determines the feature extraction capability of the algorithm and its application potential in downstream tasks. Selecting a suitable backbone can help the network focus on improving fine-grained problems. Recent studies have shown that, with advanced training settings and updated structures, convolutional neural networks can achieve comparable or better performance than the ViT without increasing computational requirements. The MogaNet, designed by Li et al. (2023), utilizes an improved convolutional macro architecture and optimization strategy to achieve faster inference speed and higher accuracy with the same number of parameters. MogaNet performs information mining and channel aggregation in pure convolutional networks and is significantly superior to current mainstream backbone networks in general image classification, image retrieval, and semantic segmentation at a lower computational cost. This success demonstrates that the MogaNet network can extract highly powerful features and has significant potential for transfer learning in downstream tasks. The selected MogaNet-L in this study consists of four stages. After the input image passes through different stages, the feature map $F_{i}\in R^{H_{i}\times W_{i}\times C_{i}}$ at different scales can be obtained. i indicates the number of phases of the backbone network. $H_{i}$ , $W_{i}$ , and $C_{i}$ represent the height, width, and number of channels of the feature map. After the input image passes through stage 1, the height and width of the feature map are reduced to one-quarter of the original size. Thereafter, after each stage, the height and width of the feature map are halved, and the number of channels is increased to four times that of the previous stage.

### 3.2 Contextual information extractor

Compared to global features, local features are abundant in the image, the correlation between features is low, and the influence of occlusion is minimal. Previous fine-grained image classification methods typically employ global features to generate large prediction regions, making it challenging for the network to discern local, detailed features in unclear objects and complex scenes. Moreover, the detection objects in the image are often mixed with the background or occupy only a small part of the image, and the local features directly obtained by the network from the local region are very limited. Since each object always exists in a specific environment or coexists with other objects, a good representation of contextual information of objects/scenes can integrate global and local features, help the network detect objects, and play a key role in fine-grained classification tasks. Based on the above analysis, a 3-layer contextual information extractor CIE is designed. Each layer has the same structure and is composed of a multi-scale sliding window and attention guide mechanism. Through a multi-scale sliding window, spatial contextual information of deep features is captured. Then, an attention-directing mechanism is used to suppress low-contextual information areas of shallow features and up-sample deep features to provide an adequate representation of important targets.

**Figure 2:**
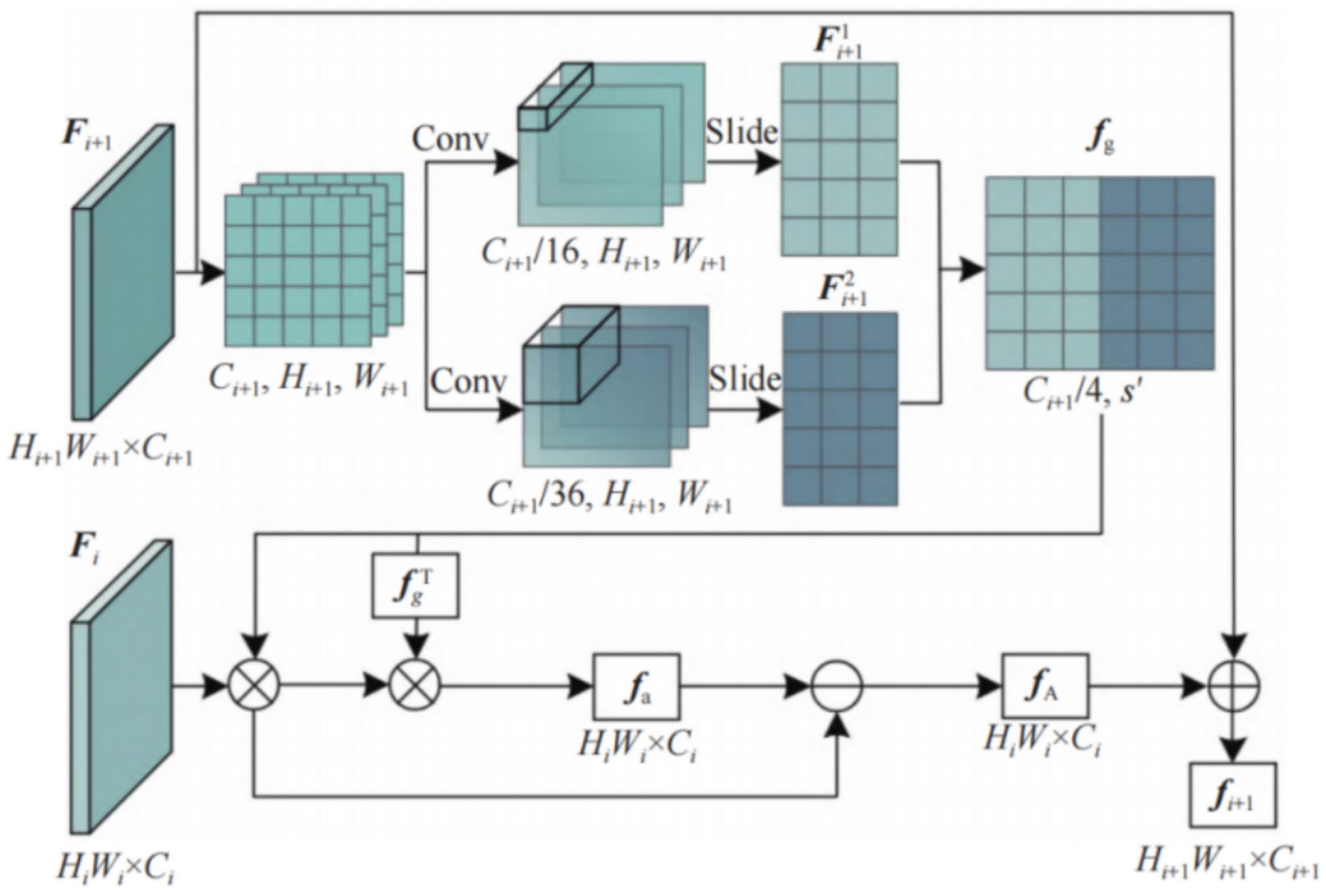
Framework of a single-layer contextual information extractor.

The single-layer structure of CIE is shown in Figure 2. Its input comes from the output feature map $F_{i}$ and $F_{i+1}$ of any adjacent layer 2 of the backbone network. $F_{i}\in R^{H_{i}\times W_{i}\times C_{i}}$ , $F_{i+1}\in R^{H_{i+1}\times W_{i+1}\times C_{i+1}}$ , where $F_{i}$ is a shallow feature and $F_{i+1}$ is a deep feature.

First, a two-dimensional convolution is used to change the number of channels of the deep feature $F_{i+1}$ into $C_{i+1}/16$ and $C_{i+1}/36$ , and the spatial contextual information of the deep feature is mined through a sliding window operation. The expression is shown in Equations 1 and 2.

(1) $$F_{i+1}^{1}=\text{Unfold}(\text{Conv}2d(F_{i+1}),s_{1})$$

(2) $$F_{i+1}^{2}=\text{Unfold}(\text{Conv}2d(F_{i+1}),s_{2})$$

Unfold shows the sliding window operation. $s_{1}$ and $s_{2}$ are the sizes of the two sliding windows, respectively. $F_{i+1}^{1}$ and $F_{i+1}^{2}$ respectively represent the feature map obtained after the sliding window operation. The number of channels of $F_{i+1}^{1}$ and $F_{i+1}^{2}$ is determined by the size of the sliding window. Set $s_{1}=2\times 2$ and $s_{2}=3\times 3$ , the number of channels of the two feature graphs after sliding window operation is $C_{i+1}/4$ , which is consistent with the number of channels $C_{i}$ of shallow feature $F_{i}$ . By concatenating $F_{i+1}^{1}$ and $F_{i+1}^{2}$ together, it can obtain the high-level descriptor $f_{g}\in R\frac{C_{i+1}}{4}\times s\prime$ , where s’ is the sum of the widths of the two features after the sliding window operation. $f_{g}$ aggregates features from multiple local parts of a deep feature and their contextual information and can emphasize the importance of the local. The $f_{g}$ is computed is as Equation 3.

(3) $$f_{g}=\text{concate}(F_{i+1}^{1},F_{i+1}^{2})$$

where concate represents the feature concatenation function.

Inspired by the attention approach, we align the high-level descriptor $f_{g}$ with the shallow feature $F_{i}$ to calculate the similarity between them and obtain the repeated contextual information $f_{a}$ in $f_{g}$ through the vector inner product operation. $f_{a}\in R^{H_{i}\times W_{i}\times C_{i}}$ . The $f_{a}$ expression is as Equation 4.

(4) $$f_{a}=(f_{g}⊕F_{i})⊕f_{g}^{T}$$

The deep layers of the network perceive more comprehensive semantic information, while the shallow layers focus on details such as edges and corners. That is, deep features have more semantic context, while shallow features have more detailed context, so for ambiguous targets in complex backgrounds, repeated contexts will contain more useless semantics. Therefore, redundant features are removed from shallow features to extract fine-grained local feature $f_{A}$ with spatial context from global information. The $f_{A}$ expression is as Equation 5.

(5) $$f_{A}=F_{i}-f_{a}$$

The feature $f_{i+1}$ containing rich contextual information is obtained by downsampling the local feature to the deep feature. $f_{i+1}\in R^{H_{i+1}\times W_{i+1}\times C_{i+1}}$ , i∈[1,3] . The expression for $f_{i+1}$ is as Equation 6.

(6) $$f_{i+1}=F_{i+1}+f_{\mathrm{fpn}}(f_{A})$$

where $f_{\mathrm{fpn}}$ represents the feature pyramid sub-sampling function.

### 3.3 Multi-level gating mechanism (MGM)

**Figure 3:**
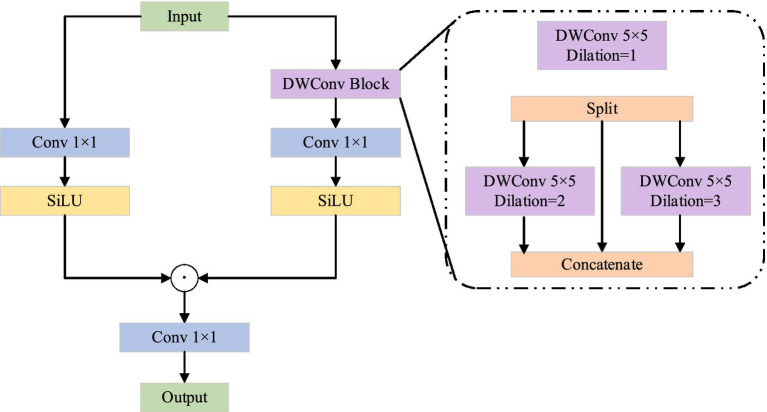
MGM structure.

The multi-level gating mechanism is derived from MogaNet, a pure convolutional neural network inspired by Vision Transformers (ViT). Its characteristic is to promote the extraction of middle-order features, thereby improving the model’s performance. The multi-level gating mechanism is shown in Figure 3. The structure of the multi-level gating mechanism consists of two branches, and the left branch can be regarded as a residual connection. The branch on the right is mainly used for feature extraction. For the feature extraction of the input feature map, a depth-separable convolution operation is first performed using a 5 × 5 convolution kernel. Then the feature graph is sliced into three sub-feature graphs. For two sub-feature graphs, convolution kernels with dimensions of 5 × 5 and 7 × 7 are used for feature extraction. In the MGM model, the design of multi-branch convolution (5 × 5, 7 × 7) is primarily adopted to capture image features at different scales, thereby better handling various structural and textural information within the image. The features in an image can be classified into fine-grained features (such as texture and detail) and coarse-grained features (such as shape and structure). Convolution kernels of different sizes can extract these features at different scales, thereby providing richer information for the model.

Dilated convolution is a special convolution operation. It expands the receptive field of the convolution kernel by inserting a dilation rate between the elements of the convolution kernel without the need to add additional parameters. This design enables the model to capture a wider range of contextual information without increasing the computational complexity.

The other one is directly mapped to the next layer, and the three sub-feature graphs are concatenated using a 1 × 1 convolution for fusion. Then, the SiLU function (Elfwing et al., 2018) is used for feature activation. Finally, the features on the two branches are added, and the output is adjusted using a 1 × 1 convolution. The advantage of a multi-level gating mechanism is that the use of large convolution kernels and expansive convolution of different sizes can significantly enhance the receptive field of the network, thereby capturing multi-level features and improving the model’s performance (Yin et al., 2024a; Yin et al., 2024b).

### 3.4 Feature elimination strategy

A fuzzy category refers to the result of predicting similar classification scores, which is one of the primary causes of misclassification. After obtaining the features containing contextual information through the above CIE, a feature elimination strategy is designed to further eliminate the influence of background and fuzzy classes on the network, allowing it to focus on more discriminative features. By eliminating similar regions and background features between classes, forcing the network to focus on other discriminant features, ViT-SAC (self-assessment classifier) examined the fuzziness in the first K prediction classes and utilized the images and the first K prediction results to reevaluate the classification. This research method is inspired by ViT-SAC, but the proposed method is different from it. (1) This method does not need to crop the original image through the generated feature map and re-input it into the network, achieving end-to-end training and avoiding the impact of training errors and errors in the previous stage on the network training in the later stage; (2) This method uses prediction scores to divide the feature map into object candidate regions and background candidate regions and deletes the corresponding fuzzy class feature points and background feature points directly at the pixel level. Since the output $f_{i+1}$ of CIE is context-sensitive, and deleting certain features does not lose the contextual information of the image.

**Figure 4:**
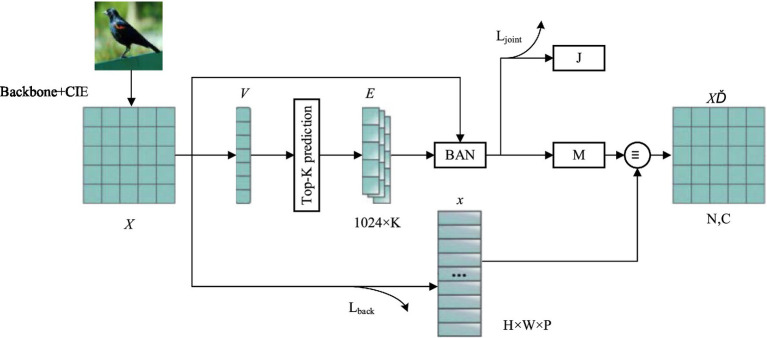
Framework of single-layer feature elimination strategy.

The feature elimination strategy (FES) consists of four modules, each with the same structure. The specific structure of a single module is shown in Figure 4. X is the input of this module. X’ represents the output of this module. Input $X^{1}$ of module 1 is the feature graph $F_{1}$ in stage 1. Inputs $X^{2}\sim X^{4}$ of modules 2–4 are outputs $f_{i+1}$ of CIE, i∈[1,3] . Each FES module contains a fuzzy class feature elimination branch and a background elimination branch. The double-branch structure comprehensively considers the contribution degree of fuzzy class features and background features to network classification. It generates discriminative features by eliminating the feature points that are less helpful to classification. For any module of FES, the feature points in the input feature graph X are numbered one by one. $X_{j}$ represents the j-th feature point in X , j∈{1,2,⋯,H×W) . The feature number set Z contains the numbers of all the feature points in X . Z can be expressed is as Equation 7.

(7) Z={1,2,⋯,H×W}

The feature elimination branch of the fuzzy class is used to eliminate the feature points of common concern of the first K terms fuzzy class. First, the visual feature $V\in R^{d_{V}}$ is extracted by the fully connected layer, and the first K prediction results are obtained, where $d_{V}$ represents the dimension of V . Then, the Glove word embedding method is used to learn the language modal information of class tags $E=[E_{1},\cdots ,E_{k},\cdots ,E_{K}]$ , $E_{k}\in R^{d_{e}}$ . This article uses the default $d_{e}$ =1,024 in the Glove to represent the dimension of each class label information. Bilinear attention network (BAN) (Kim et al., 2018) is used to fuse the two-modal information X and E , and the joint feature $J\in R^{d_{e}}$ and fuzzy attention map $M\in R^{H\times W}$ are obtained, with the expression is as Equations 8–10.

(8) m=σ(Linear(X)×E)

(9) M=sum(m).resize(H,W)

(10) $$J=\sum_{j}^{H\times W}\sum_{1}^{K}X_{j}^{T}\times M\times E_{k}$$

where σ(⋅) represents the Softmax function. Linear(⋅) indicates the fully connected layer. m represents K feature maps obtained by BAN. $m\in R^{H\times W\times K}$ . sum(⋅) means add by column, and resize(H,W) means return the feature vector of size ( H×W ). The response of M reflects the region of common concern among the first K fuzzy classes, and the higher the response of the feature points, the more likely it is to cause incorrect classification. The H×W feature points in M are sorted according to the response size, and the feature points of common concern of the first τ fuzzy classes are discarded, and the fuzzy cancellation feature number set $Z_{\max}$ is obtained. $Z_{\max}$ is a subset of the set Z of all feature numbers.

Background elimination is used to eliminate feature points with fewer classes, thereby reducing the influence of complex backgrounds on classification results. The classification score X of all feature points in x is extracted by the full connection layer, $x\in R^{H\times W\times K}$ . Each feature point is divided into class P , where P is the total number of classes. The maximum prediction probability is used as the classification score of the feature point, and a low classification score indicates that the feature point belongs to the background feature. The first δ feature points are selected according to the classification scores from high to low, and the background elimination feature number set $Z_{\min}$ is obtained. $Z_{\min}$ is a subset of the set z of all feature numbers.

Finally, it takes the intersection of the two numbered sets $Z_{\text{final}}=Z_{\min}\cap Z_{\max}$ . By gating the feature points in $Z_{\text{final}}$ , a new feature graph X’ is obtained, and the expression is as Equation 11.

(11) $$X^{'}=\{X_{j}|j\in Z_{\text{final}}\}$$

where $X^{'}\in R^{N\times C}$ , N is the total number of extracted feature numbers, and C is the number of channels in the feature map.

Since the feature number $g(Z_{\text{final}})$ in $Z_{\text{final}}$ is not fixed, to determine the classifier parameter of the X’ connection, it takes N=δ−τ . When $g(Z_{\text{final}})>N$ , delete the feature numbers with low classification scores in $Z_{\text{final}}$ until $g(Z_{\text{final}})=N$ ; When $g(Z_{\text{final}})<N$ , add the number of features with high classification scores to $Z_{\text{final}}$ from Zmax until $g(Z_{\text{final}})=N$ .

### 3.5 Feature fusion

Using single-scale feature maps for classification training, the network generates large prediction regions and cannot adequately characterize image details. Therefore, the multi-scale feature fusion layer is introduced to reduce the loss of detail features and fully mine multi-scale spatial information. After the FES strategy, four feature maps of different scales are obtained namely, $X1\prime =R^{N_{1}\times C_{1}}$ , $X2\prime =R^{N_{2}\times C_{2}}$ , $X3\prime =R^{N_{3}\times C_{3}}$ , $X4\prime =R^{N_{4}\times C_{4}}$ . First, the dimensions of four feature graphs are unified by using one-dimensional convolution with a convolution kernel size 1, and they are joined together along the channel dimension to obtain $X_{\text{final}}\in \mathrm{RN}\prime \times C_{1}$ . Here, $N^{'}=N_{1}+N_{2}+N_{3}+N_{4}$ . $X_{\text{final}}$ contains all the feature points mined by the network that are useful for classification. The selected local features are then recombined into global features that can represent the entire image through a fully connected layer. Thus, a feature graph of dimension $\mathrm{RN}\prime \times C_{1}$ can produce a prediction of dimension $R^{P}$ after passing through the fully connected layer.

### 3.6 Loss function

Due to small differences among the subclasses in the image classification task, the cross-entropy loss function alone is insufficient to completely supervise the network’s iterative learning. The loss functions designed in this study include prediction loss $L_{\text{final}}$ , background elimination loss $L_{\text{back}}$ , and fuzzy class elimination loss $L_{\text{joint}}$ and drop feature point loss $L_{\text{drop}}$ . The total loss L can be expressed is as Equation 12.

(12) $$L=L_{\text{final}}+L_{\text{back}}+L_{\text{joint}}+L_{\text{drop}}$$

$L_{\text{final}}$ is calculated by the cross-entropy loss function $L_{\mathrm{ce}}$ , which can be expressed is as Equation 13.

(13) $$L_{\text{final}}=L_{\mathrm{ce}}(X_{\text{final}},y)$$

where y indicates the sample label.

$L_{\text{back}}$ is used to oversee the background elimination branch of the FES policy. The average classification score $l^{i}\in R^{P}$ of all feature points is used to calculate the overall loss of $X^{i}$ with the Equations 14 and 15.

(14) $$l^{i}=\frac{1}{H\times W}\sum_{j=1}^{H\times W}x_{j}^{i}$$

(15) $$L_{\text{back}}=-\frac{1}{H\times W}\sum_{i=1}^{4}\sum_{p=1}^{P}y_{p}\log l_{p}^{i}$$

where $x_{j}^{i}$ represents the classification score of the feature point whose feature number is j in the i−th stage. $y_{p}$ represents the real label of the sample. $l_{p}^{i}$ represents the prediction probability that $l^{i}$ belongs to class p .

$L_{\text{joint}}$ is used to supervise the fuzzy class feature elimination branch of FES policy, which can be expressed as Equation 16.

(16) $$L_{\text{joint}}=\sum_{i=1}^{4}\sum_{p=1}^{P}y_{p}\log J_{p}^{i}$$

where $J_{p}^{i}$ represents the prediction probability that the feature vector $J^{i}$ of i−th stage belongs to class p .

$L_{\text{drop}}$ is calculated by the sum $h^{i}$ of the discarded features in the FES policy. The expression with the Equations 17 and 18.

(17) $$h^{i}=\sum_{j\in Z-Z_{\text{final}}}X_{j}^{i}$$

(18) $$L_{\text{drop}}=-\sum_{i=1}^{4}\sum_{p=1}^{P}y_{p}\log(1-h_{p}^{i})$$

where $h_{p}^{i}$ indicates that $h^{i}$ belongs to the prediction probability of class p .

## 4 Experimental results and analysis

In order to verify the effectiveness of the proposed method, the model training is carried out on four publicly available fine-grained datasets: Mini-ImageNet (Oreshkin et al., 2018), CUB-200-2011 (CUB) (Zhai and Wu, 2018), Stanford Dogs (Dogs) (Khosla et al., 2011), and Stanford Cars (Cars) (Jiang and Yin, 2023), and the experimental comparison is made with other few-shot fine-grained image classification methods. In addition, ablation experiments are performed to verify the performance advantages of individual modules, the complexity of multi-level gating mechanism modules in backbone networks is analyzed, and the spatial effect of feature visualization is demonstrated.

### 4.1 Experiment set

The standard fine-grained datasets CUB, Dogs, and Cars, and the few-shot dataset Mini-ImageNet, are used for comparison experiments. CUB is a representative bird dataset containing 11,788 images from 200 different bird species. The Cars dataset comprises 16,185 images of 196 different car brands. The Dogs dataset collects 120 different dog breeds and contains a total of 20,580 images. Mini-ImageNet is the most widely used dataset in the field of few-shot learning, comprising 100 categories with 600 images each, totaling 60,000 color images. The above datasets are shown in Table 1.

**Table 1:** Dataset information statistics.

Dataset	Total number	Training	Verification	Testing
CUB	200	130	20	50
Dogs	120	70	20	30
Cars	196	130	17	49
Mini-ImageNet	100	64	16	24

Although the data volume division varies under different tasks, in this study, we uniformly divide the numbers of the training set, validation set, and test set of these four datasets into 60:20:20%.

### 4.2 Experiment settings

This experiment was conducted on a desktop computer equipped with a GTX 1660S GPU, utilizing the PyTorch 1.10 framework and Python 3.7 version to complete the experiment. To evaluate the performance of a fine-grained image classification method, the accuracy index is used as the criterion for judgment. The model conducts 400 rounds of epoch training without using any additional data. The few-shot learning configurations are 5-way 1-shot and 5-way 5-shot. Among them, only 1 or 5 samples are extracted from each class as a support set so as to verify the classification ability of fine-grained images in the case of a few-shot sample. The image samples for training and testing are adjusted to 84 × 84 pixels. The Adam optimizer is used for model training, with a learning rate of 0.001. In the final testing phase, the classification accuracy of the test sample and its 95% confidence interval are evaluated by calculating the average of 600 randomly generated scenarios.

### 4.3 Experimental comparison

#### 4.3.1 Experimental comparison on a few-shot datasets

To evaluate the performance of the proposed method in the classification task, an experimental comparison is conducted on the Mini-ImageNet dataset, as presented in Table 2.

**Table 2:** Experimental comparative analysis on the mini-ImageNet dataset.

Model	5-way 1-shot	5-way 5-shot
Matching net (Zhang et al., 2021)	43.67	55.42
ProtoNet (Zhang L. et al., 2024)	49.53	68.31
RelationNet (Kang et al., 2021)	50.55	65.43
DN4 (Li and Wu, 2024)	51.35	71.13
RCN (Wang et al., 2023)	53.58	71.74
ICNN (Wu et al., 2023)	49.82	68.77
FFSNet (Ma et al., 2023)	52.48	68.30
LCNet-ViT-FG (Shen and Qin, 2025)	54.48	72.14
FET-FGVC (Chen et al., 2024)	56.44	72.95
dSE (Hossain et al., 2023)	59.53	77.47
Proposed	65.87	79.33

Table 2 shows the experimental results of the 5-way 1-shot and 5-way 5-shot classification tasks on the Mini-ImageNet dataset. The classification accuracy rates reach 65.87 and 79.33%, respectively. In the 1-shot setting, the accuracy performance of the proposed method is superior to other methods, which is attributed to two main factors: (1) The advantage of multi-scale feature fusion enhances the ability of information capture and (2) accelerated model adaptation to new categories by setting a multi-level gating mechanism.

To evaluate the performance of the model under low-light conditions, we use the low-light dataset for testing. These images simulate scenes at night or in low-light environments, accurately reflecting the model’s performance under such conditions. Table 3 presents the experimental results obtained under low-light conditions.

**Table 3:** Experimental comparative analysis on the low-light dataset.

Model	5-way 1-shot	5-way 5-shot
Matching net (Zhang et al., 2021)	42.37	54.81
DN4 (Li and Wu, 2024)	51.22	71.07
RCN (Wang et al., 2023)	53.45	71.68
LCNet-ViT-FG (Shen and Qin, 2025)	54.47	72.13
FET-FGVC (Chen et al., 2024)	55.89	71.78
dSE (Hossain et al., 2023)	59.28	77.16
Proposed	65.76	79.31

Medical images usually have high resolution and complex textures and structures, and the annotation cost is relatively high. Remote sensing images have extensive geographical coverage, multispectral information, and complex backgrounds. The images in these fields have significant differences in data distribution, feature space, and annotation methods. We conduct experiments using public medical image datasets (the ISIC skin lesion dataset). The dataset comprises high-resolution images of various skin lesions, accompanied by detailed and accurate annotations. We employ the transfer learning method and utilize a pre-trained model for the task of medical image classification. The result is shown in Table 4.

**Table 4:** Experimental comparative analysis on the ISIC skin lesion dataset.

Model	5-way 1-shot	5-way 5-shot
Matching net (Zhang et al., 2021)	41.22	52.91
DN4 (Li and Wu, 2024)	49.82	63.27
RCN (Wang et al., 2023)	51.83	65.69
LCNet-ViT-FG (Shen and Qin, 2025)	52.40	67.55
FET-FGVC (Chen et al., 2024)	53.81	68.65
dSE (Hossain et al., 2023)	54.39	71.84
Proposed	63.18	72.41

The experimental results show that through transfer learning. However, the results have declined somewhat compared with those of other datasets; the model can still achieve good performance in the task of medical image classification.

#### 4.3.2 Experimental comparison on other datasets

To evaluate the classification performance on fine-grained datasets, this experiment is compared with several methods. As shown in Tables 5–7, the tables present the performance of different models for the 5-way 1-shot and 5-way 5-shot classification tasks on three fine-grained datasets.

**Table 5:** Experimental comparative analysis on the CUB dataset.

Model	5-way 1-shot	5-way 5-shot
Matching net	60.17	74.68
ProtoNet	50.78	75.17
RelationNet	64.05	77.98
DN4	53.26	82.01
RCN	66.59	82.15
DLG	64.88	83.42
ICNN	67.67	83.73
FFSNet	68.41	80.75
LCNet-ViT-FG	54.65	75.97
FET-FGVC	64.63	78.74
dSE	71.87	85.35
BSNet	66.00	81.10
Proposed	73.87	87.58

**Table 6:** Experimental comparative analysis on the dogs dataset.

Model	5-way 1-shot	5-way 5-shot
Matching net	46.21	59.90
ProtoNet	40.92	61.69
RelationNe	47.46	66.31
DN4	45.84	66.44
RCN	54.40	72.76
DLG	47.88	67.18
ICNN	58.40	78.19
FFSNet	54.09	70.96
LCNet-ViT-FG	49.19	70.27
FET-FGVC	61.86	77.49
dSE	61.86	78.36
BSNet	51.17	68.71
Proposed	64.12	79.34

**Table 7:** Experimental comparative analysis on the cars dataset.

Model	5-way 1-shot	5-way 5-shot
Matching net	44.84	66.85
ProtoNet	36.65	62.25
RelationNet	46.15	68.63
DN4	61.62	89.71
RCN	61.73	89.73
DLG	62.67	89.09
ICNN	72.87	87.78
FFSNet	58.43	75.79
LCNet-ViT-FG	60.15	89.73
FET-FGVC	70.85	87.84
dSE	76.47	93.43
BSNet	54.23	73.58
Proposed	73.90	83.82

As shown in the table, in the CUB dataset, the proposed model achieves the best performance in the 1-shot task, with an accuracy that is 5.46% higher than that of the sub-optimal model FFSNet, and the accuracy is 2.23% higher than that of the sub-optimal model dSE in the 5-shot task. On the Dogs dataset, the 1-shot task and 5-shot task increase by 2.26 and 0.98%, respectively, compared with the dSE method. However, on the Cars dataset, the accuracy of the proposed model is also higher than that of most methods in the 5-way 1-shot setting; however, it is slightly insufficient in the 5-way 5-shot classification task. This may be due to the lack of local diversity in the dataset, which leads to the overlapping of local areas of identification, thereby limiting the improvement of classification performance. In summary, the proposed method’s accuracy is superior to that of other methods on fine-grained datasets.

### 4.4 Ablation experiment

To illustrate the effectiveness of each module, we conduct ablation experiments on three fine-grained datasets: CUB, Dogs, and Cars. Under the two settings of 5-way 1-shot and 5-way 5-shot, modules are added sequentially for training, and results are obtained. The evaluation index is Top-1 accuracy. The specific experimental results are shown in Tables 8–10.

**Table 8:** Ablation experiments with different modules on CUB.

Modules	5-way 1-shot	5-way 5-shot
MogaNet	66.01	81.02
MogaNet + CIE	69.08	83.52
MogaNet + CIE + MGM	72.35	86.16
MogaNet + CIE + MGM + FES	73.87	87.58

**Table 9:** Ablation experiments with different modules on dogs.

Modules	5-way 1-shot	5-way 5-shot
MogaNet	51.17	68.71
MogaNet + CIE	55.30	72.18
MogaNet + CIE + MGM	62.42	77.55
MogaNet + CIE + MGM + FES	64.12	79.34

**Table 10:** Ablation experiments with different modules on cars.

Modules	5-way 1-shot	5-way 5-shot
MogaNet	54.23	73.58
MogaNet + CIE	62.87	77.64
MogaNet + CIE + MGM	72.42	82.62
MogaNet + CIE + MGM + FES	73.90	83.82

According to the table results, in the 5-way 1-shot classification task, the accuracy of the benchmark network increases by 3.08, 4.13, and 8.64%, respectively, after the CIE method is added. Subsequently, with the introduction of the MGM method, the performance further improves by 3.27, 7.12, and 9.55% compared to the previous results. In addition, the inclusion of the FES module enhances model performance by approximately 1.5%. The same experiment is performed on the 5-way 5-shot task, achieving classification accuracies of 87.58, 79.34, and 83.82% on three fine-grained datasets, respectively.

### 4.5 Complexity analysis of the feature fusion module

In order to verify the lightness of FES, this study uses the Thop method to calculate the number of parameters and the structure calculation amount of the model, where the network structure computation refers to the number of floating-point operations per image (GFLOPI) (Meng et al., 2023) that occur during a single forward propagation when one image is input.

In this study, MogaNet is selected as the backbone network of this method. From the perspective of lightweight, compared to the ResNet series networks, the MogaNet network has a lower number of parameters and calculation amount, as shown in Table 11. It can be seen that the parameter number of MogaNet is 226,177, while the parameter number of ResNet12 is 10,737,672, indicating a significant difference in calculation amount. From the point of view of efficiency, the computing resources of few-shot learning tasks are often limited, and the lightweight MogaNet network can be used to train and reason more efficiently. After adding the proposed FES module based on MogaNet, the number of parameters increases by only 11,536 (approximately 5.1%), which is a small increase and does not significantly increase model complexity. Based on MogaNet, the calculation amount is increased by 1.56 GFLOPI/MB, which indicates that the calculation cost is lower. In summary, it demonstrates that FES can significantly enhance network performance while maintaining a minimal increase in parameters and computational requirements.

**Table 11:** Complexity comparison analysis.

Backbone	GFLOPI/MB	Parameters
MogaNet	40.49	226,177
MogaNet + FES	32.05	237,712
ResNet12	138.56	10,737,672
ResNet18	150.82	11,689,512

### 4.6 Loss function change curve

**Figure 5:**
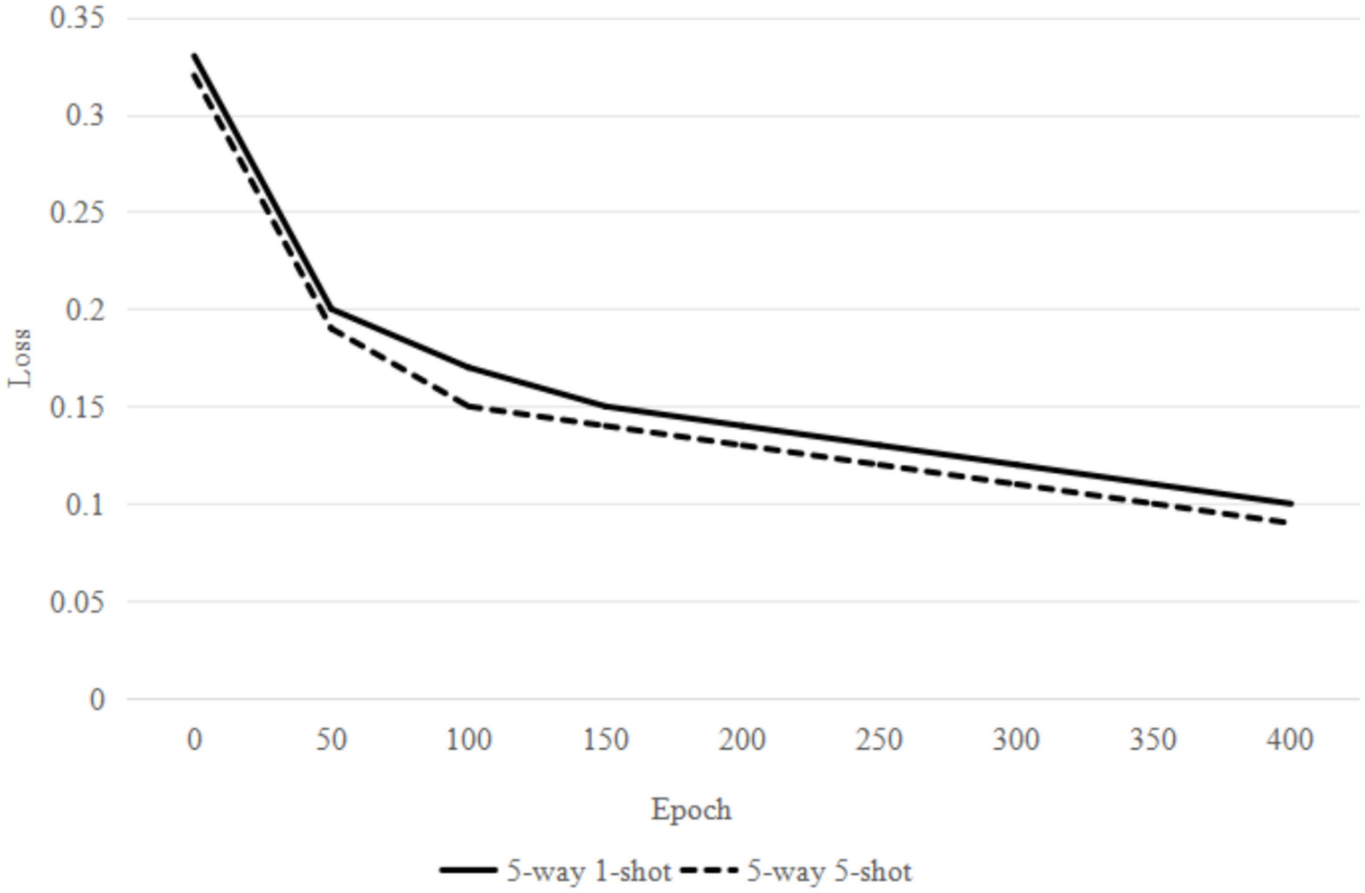
Loss change curve.

To evaluate the performance of the proposed method, loss convergence experiments are conducted on the CUB dataset for 5-way 1-shot and 5-way 5-shot settings in this section. As shown in Figure 5, the model loss changes with the increase of the training rounds under different training settings. In the first 50 rounds, the loss value of the two training settings decreases significantly, indicating that the model’s learning effect is obvious. During the intermediate period, the loss continues to decline, with the 1-shot task losing approximately 0.12 at 200 rounds and the 5-shot task losing approximately 0.1 at the same number of rounds. At this point, the model’s learning rate starts to slow down, but it continues to optimize. After 300 rounds, the loss tends to flatten out and reach a stable state. From the overall trend, the loss value of the 5-way 5-shot task is consistently lower than that of the 5-way 1-shot task, indicating that under the same number of training rounds, the model for the 5-shot task outperforms that for the 1-shot task, and its learning ability and stability are also stronger.

### 4.7 Hyperparameter analysis

In the FES model, parameters *τ* and *δ* are two key hyperparameters, which, respectively, control the sensitivity of feature extraction and the weight of feature fusion. To better understand the influence of parameters τ and δ on model performance, we conducted a parameter sensitivity analysis. We set different value ranges for the parameters τ and δ, respectively. τ∈[0.1,1.0], δ∈[0.01,0.1], and the step size is 0.05. We use accuracy to evaluate the model’s performance under different parameter settings on the CUB dataset, as shown in Tables 12, 13. It can be seen from the experimental results that when τ = 0.4 and δ = 0.08, the model yields better results.

**Table 12:** The effect of *τ* on the performance of the model.

τ	Accuracy/%
0.1	91.2
0.2	91.4
0.3	91.4
0.4	91.5
0.5	91.3

**Table 13:** The effect of δ on the performance of the model.

*δ*	Accuracy/%
0.05	92.5
0.06	92.6
0.07	92.6
0.08	92.7
0.09	92.5

### 4.8 Visualization experiment

**Figure 6:**
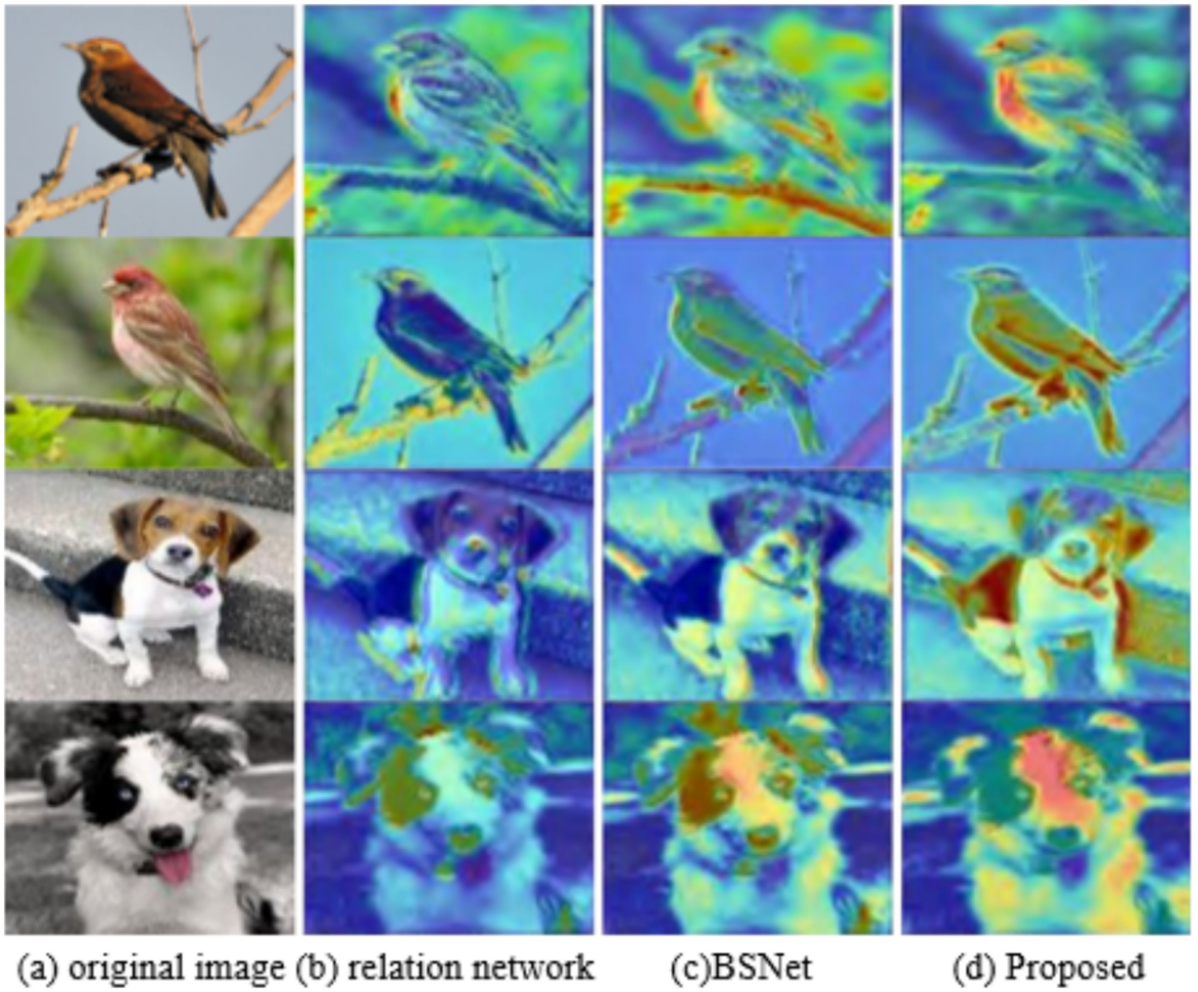
Visual result. **(a)** original image, **(b)** relation network method, **(c)** BSNet method, **(d)** proposed method.

By comparing the response region visualizations of different models, this study demonstrates the degree to which each model focuses on key features in the classification task, aiming to highlight the advantages of the model presented in this study. The original images of two bird species and two dog species are processed using the Grad-CAM algorithm. As shown in Figure 6, the proposed model in this study presents clearer and more concentrated features in the response area compared with Relation Network and BSNet. In the bird dataset, the high response area of the model is concentrated in the beak, wing, and other fine-grained features. In the dog dataset, the model responds strongly to key features of the dog, such as patterns and ears, which are crucial for distinguishing between different dog breeds. In contrast, although the response regions of Relation Network and BSNet cover some key features, the distribution is relatively scattered, lacking the high response concentration and clarity of the method presented in this study. The proposed method in this study effectively combines MGM and FES, improving the model’s response concentration in important regions and enhancing its ability to discriminate subtle image features.

## 5 Conclusion

To overcome the limitations of the traditional MogaNet model in capturing local details and the shortcomings of existing image classification methods, this study proposes a novel fine-grained image classification network. The network makes full use of the multi-scale features of the backbone to enhance its ability to capture local details. A contextual information extractor is designed, which uses contextual information and an attention mechanism to guide feature transformation, suppress low-information regions, and enhance important regions. The class prediction score is used to eliminate background features and fuzzy class features, allowing the network to focus on discriminative regions. A variety of loss functions are designed, and the constraint feature extraction and fuzzy feature elimination further improve the network’s performance. The model is evaluated on a few-shot fine-grained benchmark dataset, achieving classification accuracy superior to the majority of state-of-the-art image classification methods. In future research, the combination of transfer learning and fine-grained image recognition methods will be further explored to enhance the model’s performance across various application scenarios.

## Data Availability

The original contributions presented in the study are included in the article/supplementary material, further inquiries can be directed to the corresponding author.
